# Heteroleptic Metal Complexes of a Pyrimidinyl Based Schiff Base Ligand Incorporating 2,2′-Bipyridine Moiety: Synthesis, Characterization, and Biological Studies

**DOI:** 10.3389/fchem.2019.00862

**Published:** 2019-12-20

**Authors:** Chioma Festus, Sunday N. Okafor, Anthony C. Ekennia

**Affiliations:** ^1^Department of Chemistry, Ignatius Ajuru University of Education, Rumuolumeni, Port-Harcourt, Nigeria; ^2^Department of Pharmaceutical and Medicinal Chemistry, University of Nigeria, Nsukka, Nigeria; ^3^Department of Chemistry, Alex Ekwueme Federal University, Ndufu-Alike, Abakaliki, Nigeria

**Keywords:** pyrimidinyl, naphthoquinone, 2,2′-bipyridine, biological activities, molecular docking

## Abstract

A sequence of transition metal complexes of Mn(II), Co(II), Ni(II), and Cu(II) incorporating a novel pyrimidinyl based Schiff base ligand, 2-(4,6-dimethylpyrimidin-2-ylamino)naphthalene-1,4-dione (HL) and 2,2′-bipyridine has been synthesized and characterized using elemental, magnetic, conductance, infrared (FT-IR), nuclear magnetic resonance (^1^H- and ^13^C-NMR), electronic (UV-Vis), electrospray ionization mass spectrometry (ESI-MS), thermographic analysis (TGA), and molecular docking studies. The acquired results were consistent with the adoption of the chemical formula, [M(X)(L)(Y)]·*n*H_2_O (where M = Mn, Co, Ni, and Cu; L = Schiff base; X = 2,2′-bipy; Y = OAc; and *n* = 0,1) for the metallic complexes. HL ligand acts as a bidentate chelator and coordinates to metallic ion centre through carbonyl oxygen atom and deprotonated imide nitrogen. Similarly, 2,2′-bipy acts as a non-ionic bidentate chelator coordinating to metallic ion center via two nitrogen atoms. The mixed ligand complexes were appraised against pathogenic strains: *S. aureus, P. aeruginosa, E. coli, B. cereus, P. mirabilis, K. oxytoca, A. niger, A. flevus*, and *R. Stolonifer*. The antimicrobial studies gave moderate-good activity. Molecular docking studies on these compounds were done to indicate binding interactions between the compounds and adopted drug targets. Additionally, 1,1-diphenyl-2-picrylhydrazyl (DPPH) radical scavenging activity of the compounds were determined at different concentrations. The antioxidant study showed good radical scavenging abilities.

## Introduction

The surge in the number of drug-resistant microbial infections is persuading healthcare specialists to seek more aggressive pharmacological options. Nonetheless, the use of natural products as antimicrobial agents for treatment of microbial infections has been embraced as an effective pharmacological option. Schiff bases are one of such compounds bearing core molecular scaffolds found in natural products (da Silva et al., 2011; Hassan et al., 2013). Pyrimidine based Schiff bases have demonstrated array of biological activities owing to the presence of the imine moiety (–N = CH–) in their molecular structures and their capacity to form metal ions chelates in biological structures (Sharma et al., 2012; Ali et al., 2017; Xiqing and Zhang, 2017; Chioma et al., 2018). Examples of biologically active pyrimidinyl based compounds are 5-fluorouracil and pazopanib (5-(4-[(2,3-dimethyl-2H-indazoyl-6-yl)methylamino]-2-pyrimidinyl]amino-2-methyl benzenesulfonamide), which are adopted as antitumour agents (Osowole and Chioma, 2013; 7). Metallic complexes of pyrimidine based Schiff bases have been extensively studied because of their improved biological properties when compared to their precursor ligands as a result of chelation effect which improves the lipophilicity of the compounds. The enhanced biological actions may also be attributable to the existence of metallic ions and hetero atoms bonded to biologically active compounds (Bauer et al., 1999). They have been reported showing antimicrobial, antioxidant and antitumour properties (Satyl et al., 2004).

In addition to the improved biological properties of chelates over their precursor ligands, chelation also leads to formation of a more stable metal-organic framework. The choice of the metal connectors and the bridging ligands permits the utilization of these materials in areas, such as ion exchange, adsorption and separation processes; heterogeneous and biomimetic catalysis; sensor technologies; luminescence; drug delivery; and proton conductivity (Okasha et al., 2019). The metal-organic coordination macromolecules exhibit remarkable stability in comparison to their discrete complexes due to their macrocycle or chelate effect. 2,2′-bipyridine is a bidentate ligand with two nitrogen donor atoms that have been used expansively as a ligand in coordination chemistry and mostly in the formation of heteroleptic complexes because of its strong redox steadiness and relative easiness of functionalization (Selvaganapathy and Raman, 2016). Heteroleptic complexes encompassing 2,2′-bipyridine and other bidentate ligands have been reported as antineoplastic, cytotoxicity, antitumor, genotoxicity, and bactericidal agents (Hayder and Nesser, 2018).

We had earlier described a series of biologically active pyrimidinyl Schiff base metal complexes (Osowole and Chioma, 2016; Chioma et al., 2018; Festus et al., 2018). The improved antimicrobial and antioxidant properties of the complexes compared to the precursor ligand promoted the present research work on heteroleptic complexes of a Schiff base ligand containing an additional biologically active molecule, 2,2′-bipyridine. The geometric, electronic, magnetic, and thermal possessions of the mixed ligand compounds have been investigated using various analytical procedures. The *in vitro* antimicrobial and antioxidant activity of the complexes will be evaluated. In addition, molecular docking studies will be adopted to evaluate the molecular interactions of the compounds with some selected drug targets using “urate oxidase” from *Aspergillus favus* (PDB Code: 1WS3) and human haematopoietic cell kinase—Hck (PDB code: 2HCK) as drug targets to evaluate the antifungal and antioxidant properties of our synthesized compounds separately. Hck has been shown to play an encouraging monitoring part in mast-cell activation induced under “high-intensity” FcϵRI stimulation (Hong et al., 2007).

## Experimental Section

### Materials

The reagents; Mn^2+^(CH_3_CO_2_).4H_2_O, Ni^2+^(CH_3_CO_2_).4H_2_O, Co^2+^(CH_3_CO_2_).4H_2_O, Zn^2+^(CH_3_CO_2_).2H_2_O, 2-hydroxy-1,4-naphthoquinone (HNQ), 2-amino-4,6-dimethypyrimidine (ADMP), 2,2′-bipyridine (2,2′-bipy), dimethylsulphoxide [(CH_3_)_2_SO], methanol (CH_3_OH), acetic acid (CH_3_COOH) and trimethylamine(C_2_H_5_)_3_N stood acquired from Sigma-Aldrich.

### Instrumentations

The proton and carbon-13 NMR spectra of HL were gotten at room temperature in *d*_6_-dimethylsulphuoxide using Bruker DRX-400 MHz spectrophotometer. Chemical shifts for the NMR measurement were obtained with reference to tetramethylsilane (TMS) equally as internal standard. The electrospray ionization mass spectra (ESI-MS) fragmentation pattern of the HL was determined on micrOTOF-Q II 10390 spectrometer. The percentage carbon, hydrogen and nitrogen contents in HL and its heteroleptic divalent metallic compounds were analyzed for on Perkin-Elmer 7300 DV and Leco, CHN-932 elemental analyzers. The electronic spectral determinations were acquired in the range, 190–900 nm using Elico-SL 164 spectrophotometer, while the FTIR spectra of the synthesized compounds were acquired as triplet scans using the KBr disc method on Perkin–Elmer Fourier-Transform Infrared Spectrum BX spectrophotometer in the region 350–4,000 cm^−1^. The TGA were conducted on a SDTQ 600 thermal apparatus under the influence of a nitrogen atmosphere using an alumina pan as reference. The solid samples had weight ranges amid 10 and 12 mg with rate of heating was upheld at 10°C/min. Molar conductance (mc) measurement was obtained using HANNA HI 991300 conductivity cell meter with dip-type cell attuned in KCl solution. The metal complexes were assessed for magnetic susceptibility on a Johnson Mathey magnetic susceptibility balance at 29°C, while the diamagnetic corrections stood evaluated using Pascal's constants. Melting points for HL and its divalent metal complexes were obtained in open glass capillary tubes using Electro-thermal Temp-Mel melting point machine. All the synthesized compounds were also tested for solubility in organic solvents.

### Synthesis

#### Synthesis of 2-(4,6-dimethylpyrimidin-2-ylamino)naphthalene-1,4-dione

**Scheme 1:**
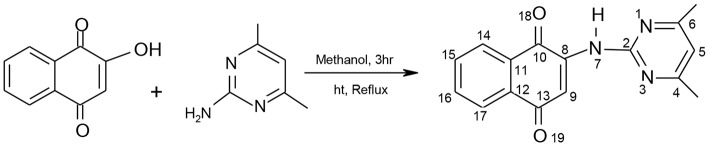
Synthetic Scheme of HL Schiff Base.

The pyrimidinyl ligand, 2-(4,6-dimethylpyrimidin-2-ylamino)naphthalene-1,4-dione (HL) was synthesized using a one-step reaction. Equimolar amounts of HNQ (2,500 mg, 0.014 mol) dissolved in 15 mL of dehydrated CH_3_OH was added to a warm solution of ADMP (1,768 mg, 0.014 mol) according to Scheme 1. The resultant combination stood refluxed for 3 h. Upon cooling on ice to 29°C, brown precipitates were obtained, filtered, recrystallized from absolute methyl alcohol and stored *in vacuo* over silica gel.

Yield = 500 mg, 68.15%. mp: 214-216°C; elemental (%)-Exp.l (Cal.d): C, 69.03 (68.80), H, 4.77 (4.69), N, 15.12, (15.05); FTIR(ν/cm^−1^): 3539_b_ (NH), 1678s (C=O), 1641s (C=N), 1652s, (C=C), 1579s (C-N), 1459s (C-C), 1384s (C-O), 982s (δC-H); UV/visible (cm^−1^): 33578, 30030 (π→π*), 26247, 22182 (n→π*); ^1^H-NMR [300 MHz, (CH_3_)_2_SO-*d*_6_] δ ppm: 3.38 (s, NH), 2.49 (s, 6H, CH_3_); 6.28 (*s*, 1H, H_5_); 7.92–7.98 (*d*, 1H, H_15, 16_); 7.76–7.79 (*s*, 1H, H_9_); 7.80–7.83 (*d*, 1H, H_14, 17_); 7.64–7.66 (*d*, 1H, H_15_); ^13^C-NMR [75 MHz, (CH_3_)_2_SO-*d*_6_] δ ppm: 159.6 (C_10_); 111.0 (C_11_); 181.3 (C_12_); 184.7 (C_13_); 131.9 (C_14_); 130.6 (C_15_); 125.4–125.9 (C_16, 17_); and 156.9 (C_2_); 106.7 (C_5_); 159.6 (C_4, 6_); 39.08–40.04 (C_20, 21_).

#### Synthesis of Divalent Heteroleptic Metallic Complexes

About, 450 mg (0.015 mol) of HL was liquefied in 20 mL of CH_3_OH; and equimolar concentrations of the divalent metal acetate and 2,2′-bipy each were added gradually to it as it stirred at 50–60°C. The resultant homogeneous solution was subsequently buffered with 0.3 mL of (C_2_H_5_)_3_N to a pH of 9 and refluxed for 5 h. The formed precipitates were permitted to cool to 29°C, filtered, washed with CH_3_OH, and kept over silica gel. All synthesized divalent metal complexes were unchanging at room temperature, non-hygroscopic and practically solvable in (CH_3_)_2_NCHO and (CH_3_)_2_SO.

##### [Mn(X)(L)(Y)]

Yield: 440 mg, 75.22%; mp: 230–232°C; elemental (%): Exp.l (Cal.d): C, 70.55 (70.43); H, 4.31 (4.23); N, 12.83 (12.77); %metal (Theo) 10.28 (10.02); μ_*eff*_(B.M.): 6.02; mw(g/mol): 548.444; color: Black; FTIR(ν/cm^−1^): 1673s (C=O), 1628s (C=N), 1583s (C=C), 1588_S_ (C-N), 1442s (C-C), 1329s (C-O), 976m (δC-H), 534s (Mn-N), 493s (Mn-O); UV-Vis (cm^−1^): 46082 (C.T), 38167, 32719 (π→π*), 25761 (n→π*), 21368 (^6^${\text{A}}_{1\text{g}}\to^{4}$T_1g_), 16077 (^6^${\text{A}}_{1\text{g}}\to^{4}$T_2g_), 12658 (^6^${\text{A}}_{1\text{g}}\to^{4}$E_g_), mc (ohm^−1^ mol^−1^ cm^2^): 5.62.

##### [Co(X)(L)(Y)]

Yield: 447 mg, 84.34%; mp: 302–304°C; elemental (%):Exp.l (Cal.d): C, 70.09 (69.92); H, 4.22 (4.19); N, 12.73 (12.68); % metal (Theo) 10.88 (10.67); μ_*eff*_(B.M.): 4.99; mw(g/mol): 552.442; color: wine-red; FTIR(ν/cm^−1^): 1669s (C=O), 1626s (C=N), 1581s (C=C), 1558s (C-N), 1475s (C-C), 1269s (C-O), 994s (δC-H), 539s (Co-N), 450s (Co-O); UV-Vis (cm^−1^): 38023, 32835 (π→π*), 25012 (n→π*), 18382 (^4^${\text{T}}_{1\text{g}}\to^{4}$T_1g_), 12642 (^4^${\text{T}}_{1\text{g}}\to^{4}$A_2g_), 11080 [^4^${\text{T}}_{1}\to^{4}$T_1g_(P)]; mc (ohm^−1^ mol^−1^ cm^2^): 8.96.

##### [Ni(X)(L)(Y)]

Yield: 446 mg, 76.90%; mp: 321–323°C; elemental (%):Exp.l (Cal.d):C, 69.99 (69.95); H, 4.23 (4.19); N, 12.70 (12.67); % metal (Theo) 10.85 (10.63); μ_*eff*_ (B.M.): 3.16; mw(g/mol): 552.224; color: maroon red; FTIR(ν/cm^−1^): 1671s (C=O), 1625s (C=N), 1590s (C=C), 1559s (C-N), 1476s (C-C),1279s (C-O), 994s (δC-H), 501s (Ni-N), 423s (Ni-O); UV-Vis(cm^−1^): 36900 (π→π*), 25356 (n→π*), 18018 [^3^A_2g_ (F)→^3^T_2g_(F)], 16159 (^3^A_2g_(F)→^3^T_1g_ (F), 12830 (^3^A_2g_(F)→^3^T_1g_ (P); mc (ohm^−1^ mol^−1^ cm^2^): 8.32.

##### [Cu(X)(L)(Y)]·H_2_O

Yield: 359 mg, 73.33%; mp: 272–274°C; elemental(%):Exp.l (Cal.d): C, 64.09 (64.04); H, 4.51 (4.39); N, 12.24 (12.18); % metal (Theo) 11.19 (11.04); μ_*eff*_ (B.M.): 1.82; mw(g/mol): 575.07; color: Dark brown; FTIR(ν/cm^−1^): 3434b (OH), 1642s (C=O), 1629s (C=N), 1590 _m_ (C=C), 1542s (C-N), 1445 _m_ (C-C), 1273s (C-O), 976 _m_ (δC-H), 551 _m_ (Cu-N), 490_m_ (Ni-O); UV-Vis (cm^−1^): 44642 (C.T), 33112, 31949 (π→π*), 26296 (n→π*), 21413 (^2^${\text{E}}_{\text{g}}\to^{2}$T_2g_); mc (ohm^−1^ mol^−1^ cm^2^): 15.2.

### Applied Studies

#### Antibacterial Studies

The antibacterial studies were carried out on HL and its heteroleptic divalent metal complexes using *B. cereus, S. aureus, K. oxytoca, E. coli, P. mirabilis*, and *P. aeruginosa* microbes which were clinical isolates acquired from microbiology laboratory, University of Ibadan, Nigeria. Nutrient agar medium was used to grow the microbial organisms for 24 h at 35°C adopting the agar well-diffusion practice (Bauer et al., 1999; Festus et al., 2018). The surfaces of the Muller Hinton's agar in petri plates were homogeneously injected with 0.2 mL of the 24 h grown test microbial cultures with the aid of pasteurized cotton swabs. After which, wells were dug into the solidified agar using a sterile cork borer (7 mm). Accordingly, 10 mg/mL solution of each test compound in dimethylsulphuroxide was poured into the well-bored. The plates were allowed to stand on the bench for 30 min before incubation at 35°C for 24 h. The actions of the compounds were evaluated using inhibition zones growth in diameter (mm), while the bactericide 1-cyclopropyl-6-floro-1,4-dihydrido-4-oxo-7-(1-piperazinyl)-3-quinoline carboxylic acid (ciprofloxacin), a second generation fluoroquinolone was used as reference drug.

#### Antifungal Studies

The *in vitro* antifungal actions of the synthesized compounds were measured using disc technique. Unpeeled but washed-sliced potatoes (250 g), dextrose (25 g), and agar (25 g) in 1,250 mL distilled water were used to prepare the ‘potato dextrose agar (PDA) media' adopted for the antifungal screening. The antifungal screening (*in vitro*) was carried out against *A. niger, A. flevus*, and *R. Stolonifer*. The pure cultures of fungal isolates were uniformly inoculated on the surface of the PDA solution petri dish. 15 μg of the stock solutions of each test sample (1 mg/mL) prepared by liquefying 10 mg of each compound in 10 mL of dimethylsulphoxide solvent was poured into a 7 mm well-bored on the PDA with a 7 mm sterile metallic cork borer. All the plates inoculated were incubated at 35°C for 48 h after which inhibition zone growth in diameter (mm) was obtained as the sensitivities of fungal isolates toward the test compounds with antibiogram zone scale. All antifungal activities were determined as mean of three replicates. The drug diflucan (fluconazole) was used as standard, while dimethylsulphoxide was used as a negative control and all values <7mm were considered inactive.

#### DPPH Radical Scavenging Studies

DPPH assay method was adopted for the *in-vitro* free radical scavenging activities of HL and its heteroleptic divalent metallic compounds. A blank solution of DPPH (100 μL) prepared in 3.0 ml of dimethylsulphoxide was examined for initial absorbance at 516 nm using UV–VIS spectrophotometer. Separately, a 50, 100, and 200 μg/mL of all synthesized compounds and the standard (ascorbic acid, 0.1 mL) made in dimethylsulphoxide were combined with 2.9 mL of DPPH (0.025 μg/mL). Test tubes containing reaction mixtures were shaken energetically, kept in the dim for 30 min and allowed to equilibrate at 29°C. The absorbance of each reaction mixture was recorded at 515 nm against the blank. All experiments were three times determined. The expression below was adopted for the evaluation of percentage scavenging ability of the DPPH radical.

$$\begin{array}{l} \%\text{DPPH scavenging influence }\left(\%\right)=\frac{\left(Ac-As\right)\times 100}{Ac} \end{array}$$

where *Ac* = Absorbance of blank and *As* = Absorbance of test sample/compound or the standard antioxidant (ascorbic acid).

### Molecular Docking Studies

**Figure 1:**
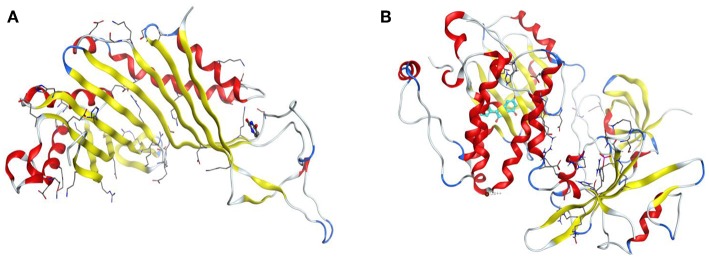
3D Structures of urate oxidase and human haematopoietic cell kinase. **(A)** 1WS3. **(B)** 2HCK.

The drug targets, urate oxidase from *Aspergillus favus* (PDB Code: 1WS3) and human haematopoietic cell kinase—Hck (PDB code: 2HCK) were adopted for appraisal of the antifungal and antioxidant properties of the synthesized compounds independently. Figure 1 below displays the 3D structures of urate oxidase and human haematopoietic cell kinase.

**Scheme 2:**
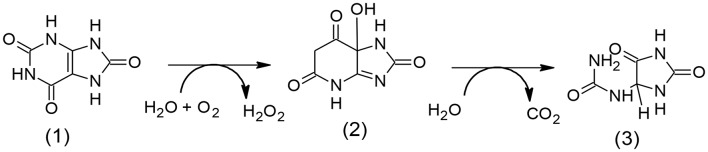
Graphic degradation pathway of uric acid (1) to allantoin (3) through (2) (5-hydroxyisourate).

The Urate oxidase (uricase; EC 1.7.3.3) is an indispensable enzyme accountable for the initial step of the degradation of uric acid into allantoin (Scheme 2).

## Results and Discussion

A first-hand sequence of heteroleptic compounds of cobalt (II), nickel (II), copper (II), and manganese (II) with a unique pyrimidinyl based Schiff base, 2-(4,6-dimethylpyrimidin-2-ylamino)naphthalene-1,4-dione (HL) as a principal chelator and 2,2′-bipy as a subordinate chelator has been synthesized. All the desired complexes were prepared in 1:1 ratio in methyl alcohol solution. The structures of the Schiff base as well as its divalent heteroleptic metallic compounds stood established on the source of experimental, spectral and magnetic records. The metallic compounds were strongly colored and stable solids.

### Elemental, Percentage Metal, and mc Analyses

The results obtained from the elemental and percentage metal analysis stood excellent in correlation with their theoretical data for coordination of HL and 2, 2'-bipy to the divalent metals in 1:1:1 stoichiometry. Generally, the heteroleptic divalent complexes were stable at atmospheric conditions and displayed various colors. The mc measurements for the complexes evaluated in (CH_3_)_2_SO had values within 5.62–15.2 ohm^−1^ cm^2^ mol^−1^ displaying non-electrolytic nature. The values designates electrolytes in (CH_3_)_2_SO are beyond 23 and 90 ohm^−1^ cm^2^ mol^−1^ for 1:1 and 2:1 electrolytes, respectively (Geary, 1971; Frisch, 2009).

### ESI-MS Studies

**Scheme 3:**
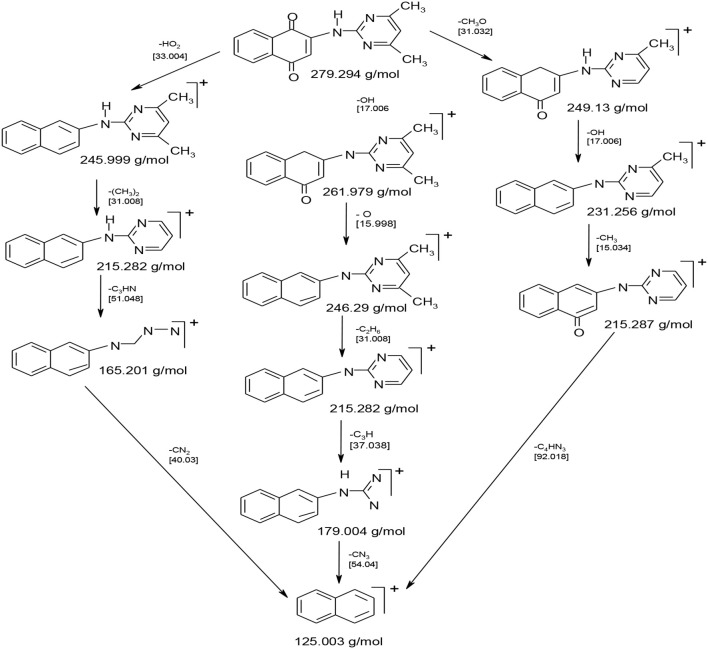
Fragmentation pattern of HL.

The ESI-M spectrum of HL (**Figure 4**) with the molecular formula, C_16_H_13_N_3_O_2_ suggested fragmentation pattern and stoichiometric compositions for HL (Scheme 3). The spectrum displayed molecular ion peak in agreement with the elemental compositions suggested by CHN analysis result and TGA and DTA fragmentations. The observed molecular ion peak (m^+^.) at m/z 278 was due to (L)^+^ loss of H that corresponds to the molecular weight (279.294 g/mol) of HL. Generally, multiple ion peaks representing three fragmentation pathways of HL and the formation of different fragments were observed in the ESI-mass spectrum corroborating the reaction of HNQ and ADMP to form HL. Besides the molecular ion peak, HL displayed fragment ion peaks at m/z 261.979, 249.13, 245.999, 215.287, and 165.201 that corresponds to loss of [OH; m/z = 17.096]^+^, [CH_3_O; m/z = 30.164]^+^, [HO_2_; m/z = 33.204]^+^, [C_2_H_6_; m/z = 31.068]^+^, and [C_3_NH_3_; m/z = 51.007]^+^, respectively. The peaks at *m/z* 246.29, 321.26, 215.282, 179.004, and 124.21 were due to loss of O, OH; CH_3_ and C_2_H_6_; C_3_H; CN_2_, CN_3_, and C_4_HN_3_ moieties. Also, L+2 peak at *m/z* 281.295 was displayed. The less intensity peak detected at *m/z* 282.937 can be ascribed to additional mass units, a consequence of C-13 existence while the intermediate peak at 276.73 may be owing to loss of hydrogen atoms. Scheme 3 displays the ESI-M spectrum plus the disintegration pathways of HL, respectively.

### ^1^H- and ^13^C- Nuclear Magnetic Resonance Spectral Analysis

The ^1^HNMR is often employed to identify tautomers of amino derivatives of naphthoquinone usually present in solutions. The proton NMR spectrum for HL obtained in (CH_3_)_2_SO-*d*_6_ showed non-existence of OH peak characteristic of HNQ around 17.0–12.0 ppm (Cherayath et al., 1990). The observation stood apportioned to the existence of ketoamine tautomeric isomer of HL in solution rather than the enolimine tautomer giving credence to an aminonaphthoquinone ligand formation (Ekennia et al., 2019). The proposed HL structure (Scheme 1) was further supported by the appearance of a broad-like singlet peak at 3.38 ppm consistent of an NH moiety. The naphthalene cyclic hydrogens were detected at the ranges 7.76–7.98 ppm as multiplets, 7.35 ppm as singlet while the pyrimidine proton was seen as a singlet at 6.28 ppm. Also, the methyl protons were observed as sharp singlet peak at 2.49 ppm. The ^13^C NMR spectrum of HL showed resonance signals common of the napthoquinone carbon atoms (C_10_, C_11_, C_12_, C_13_, C_14_, C_15_, C_16_,_17_, C_18_, and C_19_) at 159.6, 111.0, 181.3, 184.7, 131.9, 130.6, 125.4–125.9, 133.2, and 134.5 ppm. The signal at the range 39.08–40.04 ppm was typical of the methyl (CH_3_) substituent carbon atoms. Furthermore, signals at 156.9, 106.7, and 159.6 ppm were accredited to C_2_, C_5_, and C_4_,_6_ atoms, respectively, of the pyrimidine.

### FTIR Spectra Studies

Relevant infrared spectral bands of the synthesized compounds were tentatively assigned by comparison with literature reports on similar compounds of related functional groups (Kumar et al., 2013). The medium band at 3,539 cm^−1^ in the HL spectrum was apportioned to the stretching vibration of a secondary amine group, N-H (Halli et al., 2004). The N-H band was completely lacking in the spectra of the complexes, an indication that coordination of HL to the metal ions took place through deprotonated amine nitrogen group. The band at 3,584 cm^−1^ in the spectrum of the divalent copper complex was attributed to vibration of coordinated water molecules. Infrared spectra of enolimine aminopyrimidinyl Schiff base complexes display three basic regions of concern within 1,650–1,550, 1,550–1,450, and 650–500 cm^−1^ typical of ν(C=N) of imine moiety, ν(C-N) of the aromatic –C-N- ring and the ν(M-N) group (Ekennia et al., 2019). Similarly, the band at 1,678 cm^−1^ in the spectrum of HL consistent of ν(C=O) underwent lower wave number (1,673–1,642 cm^−1^) shifts in the divalent complexes, corroborating the participation of the ketonic oxygen atom in coordination to the metallic ions. The new bands detected in the complexes within the regions 501–551 and 493–421 cm^−1^ were apportioned to ν(M-N) and ν(M-O) vibrations separately.

### UV-Vis Spectra Studies

The ultraviolet spectra of the synthesized compounds showed n →π* and π→π*; and *d*-*d*/L→MCT. The HL displayed two absorption bands in its ultraviolet spectrum around 32,362 and 29,019 cm^−1^ typical of n→π* and π→π* transitions. The intra-ligand (*d*-*d*/L→MCT) bands were detected at lesser frequencies in the spectra of the heteroleptic divalent complexes, an outcome of coordination of HL and 2,2′-bipy to the divalent metallic ions.

The ultraviolet spectrum of the synthesized divalent manganese complexes exhibited three absorptions around 25,761, 32,719–38,167, and 46,082 cm^−1^, consistent of n→π*, π→π* and charge transfer transitions. Divalent manganese complexes are largely high spin, with their spectra often characterized by parity forbidden transitions resulting from weak spin. The transitions are often accredited to the existence of a ^6^S lower term and higher foursome (^4^G) state. The transitions ^6^${\text{A}}_{1\text{g}}\to^{4}$T_1g_, ^6^${\text{A}}_{1\text{g}}\to^{4}$E_g_(G), and ^6^${\text{A}}_{1\text{g}}\to^{4}$T_2g_(G) arising from three weak absorption bands are commonly used to characterize divalent manganese complexes in the octahedral field (Abu-el-wafa et al., 1989). The synthesized heteroleptic manganese complex displayed visible bands at 21,368, 16,077, and 12,658 cm^−1^ consistent of ^6^${\text{A}}_{1\text{g}}\to^{4}$T_1g_(ν_1_), ^6^${\text{A}}_{1\text{g}}\to^{4}$T_2g_(G) (ν_2_), and ^6^${\text{A}}_{1\text{g}}\to^{4}$E_g_(G) (ν_3_) transitions and typical of octahedral geometry (Figgis, 1966; Abdou et al., 2015). Experimental magnetic moment figure of 6.02 B.M corroborated the assigned high spin octahedral geometry since divalent manganese complexes of 3*d*^5^ electron configuration exhibits magnetic moments (5.92 B.M) equivalent to five un-combined electrons (Cotton et al., 2003).

The uv-vis spectrum of the heteroleptic cobalt complex displayed absorption bands around 28,023, 32,835, and 52,012 cm^−1^. The bands at 28,023 and 32,835 cm^−1^ aroused from *n*→π* and π→π* transitions of the C=O, C = N, and C = C moieties in the naphthoquinone, pyrimidine, and 2,2′-bipy rings of the ligands, while the band at 52,012 cm^−1^ is consistent with intra complex charger transfer transition (Sharma et al., 2012). Additionally, *d-d* bands were observed around 18,382, 12,642, and 11,080 cm^−1^ corroborating ^4^${\text{T}}_{1\text{g}}\to^{4}$T_2g_, ^4^${\text{T}}_{1\text{g}}\to^{4}$A_2g_ and ^4^${\text{T}}_{1\text{g}}\to^{4}$T_1g_(p) transitions of an octahedral d^7^ (high spin) geometry through ^4^F ground term (Raman et al., 2004; Osowole et al., 2008). The assignment of high spin octahedral geometry to the divalent heteroleptic cobalt complex was verified with the calculated magnetic moment value of 4.99 BM. Low spin octahedral cobalt complexes often display magnetic values between 1.82 and 2.22 BM, while high spin octahedral cobalt complexes display magnetic moment in the range 4.60–6.05 BM (Abd El-Wahed et al., 2008).

The heteroleptic nickel complex under current examination showed three bands in the regions 11,619, 18,018, and 23,632 cm^−1^. These bands were assigned to ^3^${\text{A}}_{2\text{g}}\to^{3}$T_2g_, ^3^${\text{A}}_{2\text{g}}\to^{3}$T_1g_, and ^3^${\text{A}}_{2\text{g}}\to^{3}$T_1g_(P) transitions, in an octahedral environment. The band at 11,619 cm^−1^ was recalculated with a band fitting procedure (Underhill and Billing, 1966). Four coordinate divalent nickel tetrahedral complexes are usually paramagnetic in nature with magnetic moments values of 3.20–4.20 B.M (Chioma et al., 2018). The octahedral geometry assigned to the synthesized nickel complex was validated by the value of 3.16 B.M obtained from the magnetic susceptibility measurement which also conforms to high spin system since six coordinate nickel complexes often display magnetic moments within 2.90–3.30 BM. The ligand field parameters (Racah inter electronic repulsion parameter, ligand field splitting energy, and ligand field stabilization energy) further corroborates the assigned six coordinate octahedral geometry (Satyanarayana, 2001). Apparently, the bands at 36,900 and 25,356 cm^−1^ observed in the ultraviolet spectrum of the synthesized heteroleptic nickel complex were assigned to π→π* and *n*→π* transitions.

The visible spectrum of the synthesized divalent copper complex presented a single broad absorption band in the visible region around 21,413 cm^−1^ consistent with ^2^E→^2^T_2_ transition and typical of an octahedral geometry of an elongated/distorted six coordinate field (Lever, 1984; Cotton et al., 2003). The three bands observed at 44,642, 33,112–31,949, and 26,296 cm^−1^ are typical of charge transfer, π→π* and *n*→π* transitions. Magnetic moment values of divalent copper complexes are usually not employed for geometry prediction alone but also for information regarding the number of metal centers in a complex. Mononuclear divalent copper complexes are expected to exhibit moment values of 1.9–2.2 B.M regardless of geometry, while dinuclear divalent copper complexes may have higher values (Khalil et al., 2002). Considering spin-orbit coupling and orbital contributions, the effective magnetic data of divalent copper assemblages at room temperature are frequently greater compared to spin-only figure of 1.73 B.M. So the studied divalent copper complex had magnetic moment of 1.86 B.M validating its mononuclear nature (Ceyhan et al., 2011) and the occurrence of an unpaired electron on the copper ion in a d^9^ configuration (Estes et al., 1978).

### Thermal Analysis

This study utilized thermal gravimetric (TG) and differential scanning calorimetry (DSC) to evaluate the synthesized compounds and are contained in Table 1. The HL in its thermo-gram indicated a melting point initiated at the temperature 195°C with a peak melting temperature at 220°C, substantiating the obtained experimental value (214–216°C). Generally, the metallic compounds in their respective thermo-grams presented two phases of disintegrations at dissimilar temperature ranges. Percentage mass losses experimentally obtained were observed to be in close agreement with the values theoretically obtained as percentage mass losses. The thermo-gram of the divalent heteroleptic manganese compound displayed the integration of the complex amid 240–285°C with corresponding loss of CO as gas with OCH_3_ group. This was immediately accompanied by another decomposition stage within 445–480°C indicating the release of the gas; NO, as well as the loss of the organic functions, OC_2_H_6_ and C_10_H_8_N. At 645°C, the thermo-gram displayed a decomposition curve consistent of the residue Mn and organic fragments, C_14_H_6_N_3_. The divalent cobalt complex in its thermo-gram revealed the disintegration of the complex within 290–340°C, showing the loss of OCH_3_ moiety and liberation of one mole each of carbon mono oxide (CO) and nitrogen dioxide (NO_2_) as gases. The second stage ensued at 350–600°C with the loss of the organic group, C_10_H_8;_ liberation of the gas, NO as well as loss of OCH_3_ functional group. The disintegration remainder from 610 to 650°C was the organic assemblage, C_14_H_6_N_2_ with Co residue. The thermo-gram of the divalent heteroleptic nickel compound indicated that the decomposition began at 240–310°C temperature range with the loss of a methoxyl (OCH_3_) molecule as well as carbon monoxide (CO) gas. This was immediately followed by the release of the gas, N_2_O within 310–340°C, while the organic moieties, C_10_H_8_N and OCH_3_ were lost at the temperature range, 340–460°C. The decomposition fragments were observed as Ni residue and C_14_H_6_N_2_ organic moiety. The Cu(II) complex initiated its decomposition as observed in the thermo-gram from 250 to 320°C with the liberation of H_2_O and OCH_3_ moieties with the release of the gases, 2NO and CO. The organic groups OCH_3_ and C_8_H_10_ were lost within the temperature 320–450°C forming the second decomposition stage. The Cu residue as well as the carbon-based molecule (C_14_H_6_N_3_) were noticed as remainders.

**Table 1:** Thermal data presentation of HL and its complexes.

**Compound**	**Temperature Range (^**o**^C)**	**% Exp. Mass loss** **(theoretical mass loss)**	**Interpretation**
HL	195–220	–	Melting point
[Mn(X)(L)(Y)] Residue	240–285 445–480	10.65 (10.79) 40.00 (39.79) 49.34 (49.52)	OCH_3_ + CO NO + OC_2_H_6_ + C_10_H_8_ Mn + C_14_H_6_N_3_
[Co(X)(L)(Y)] Residue	290–340 350–600	18.50 (18.65) 35.0 (36.97) 46.50 (47.35)	N_2_O + OCH_3_ + CO OC_2_H_6_ + C_10_H_8_ + NO Co + C_14_H_6_N_2_
[Ni(X)(L)(Y)] Residue	240–310 310–460	11.0 (10.72) 42.0 (42.05) 47.0 (47.32)	CO + OCH_3_ N_2_O + C_10_H_6_N + OC_2_H_6_ Ni + C_14_H_6_N_2_
[Cu(X)(L)(Y)].H_2_O Residue	250–320 320–450	21.0 (21.04) 30.0 (30.30) 49.0 (48.72)	H_2_O +CO+2NO + OCH_3_ OC_2_H_6_ + C_10_H_8_ Cu + C_14_H_6_N_3_

### Antibacterial Studies

The antibacterial results of the HL, 2,2′-bipy and their heteroleptic divalent Mn, Co, Ni, and Cu complexes (Table 2) indicate that the compounds generally had activity against all the microorganisms, except Ni(II) complex which showed no sensitivity against *K. oxytoca* and *P. aureginosa*. As expected, the divalent metallic compounds had significant sensitivity than HL and 2,2′-bipy. For instances, all metallic compounds exhibited excellent activities than that of the ligands against the bacteria *E. coli* and *P. mirabilis* with inhibitory growth zones within 18.0–28.0 and 18.5–28.0 mm. Furthermore, divalent manganese, cobalt, and copper complexes unpredictably demonstrated enhanced susceptibility than HL and 2,2′-bipy against *B. cereus* with inhibitory growth regions of 31.0, 29.0, and 29.0 mm separately. Similarly, the trio complexes displayed broad-spectrum actions greater than that of both the ligands and the standard drug against *P. aeruginosa* (31.0 mm), *S. aureus* (34.0 mm), and *P. mirabilis* (27.0 mm); *P. aeruginosa* (32.0 mm) and *P. mirabilis* (26.0 mm); and *P. mirabilis* (28.0 mm) proving their potential usefulness as broad-spectrum antibacterial agents. The improved activities of the divalent complexes may be explained on the hyper conjugation of the coordinated aromatic ring systems and induced higher liposolubilibity (Ekennia et al., 2019), a consequence of the decreased metal ions polarity leading to the delocalization of π-electrons over the heteroleptic complex assemblage (Al-Amiery et al., 2012). The delocalization of π-electrons also favored penetration of the divalent compounds through the phospholipid layers of the bacterial membrane thereby enhancing antimicrobial activity (Ajibade and Zulu, 2011; Al-Amiery et al., 2012). Additionally, chelation neutralizes several cellular enzymes, crucial for metabolic activities in the microbes (Abuo–Melha and Faruk, 2008). The divalent cobalt complex exhibited the best antibacterial action comparing positively to the activity of the standard drug, against the microorganisms making the compound suitable for antibiotic drug research interest in the close future (Ajibade and Zulu, 2011; Osowole and Ott, 2012).

**Table 2:** Antibacterial data of HL, 2,2′-bipy and their heteroleptic complexes.

**Compound/bacteria**	***B. cereus***	***E. coli***	***K. oxytoca***	***P. aeruginosa***	***S. aureus***	***P. mirabilis***
HL	24.5	16.0	26.0	18.0	11.0	16.0
Bipy	15.5	12.0	26.0	8.5.0	17.0	19.0
[Mn(X)(L)(Y)]	31.0	18.7.0	23.0	16.0	20.0	28.0
[Co(X)(L)(Y)]	29.0	21.0	27.0	32.0	23.0	26.0
[Ni(X)(L)(Y)]	21.0	28.5.0	R	R	26.0	18.0
[Cu(X)(L)(Y)].H_2_O	29.0	22.0	24.0	31.0	34.0	27.0
^+^Ciprofloxacin	33.0	32.0	36.0	26.0	29.0	23.0
-(CH_3_)_2_SO	R	R	R	R	R	R

### Antifungal Results

The results of the antifungal activity of the synthesized HL, 2,2′-bipy and their heteroleptic divalent complexes (Table 3) against *A. niger, A. flevus, and R. Stolonifer* specify that theyo exhibited good to moderate inhibition growth zones. The fungal microbes were generally inhibited by the ligands with higher inhibitory zones of 13.0–29.0 mm. The actions of the ligands against the fungal spieces were unpredictably higher than that of the divalent metallic complexes (Nogrady, 1988). However, [Co(X)(L)(Y)] exhibited comparable activity to that of the starting ligands against *A. niger* (23.0 mm), *R. Stolonifer* (25.0 mm), and *A. flevus* (21.0 mm), a consequence of the well-absorption of divalent cobalt ions on the surface of the fungal cell walls.

**Table 3:** Antifungal data of HL, 2,2′-bipy and its heteroleptic complexes.

**Fungal/compounds**	***A. niger***	***A. flevus***	***R. Stolonifer***
HL	23.0	29.0	27.0
Bipy	16.0	19.0	13.0
[Mn(X)(L)(Y)]	13.0	21.0	–
[Co(X)(L)(Y)]	23.0	21.0	25.0
[Ni(X)(L)(Y)]	13.0	23.0	–
[Cu(X)(L)(Y)].H_2_O	–	–	–
^+^ Fluconazole	36.0	29.0	38.0
-(CH_3_)_2_SO	–	–	–

### DPPH Radical Scavenging Studies

DPPH, a radical scavenger is a dependable and standard assay for antioxidant capability evaluations. The HL, 2,2′bipy and the heteroleptic divalent complexes were evaluated for free radical scavenging capability with DPPH radical at 50, 100, and 200 μg/mL in 1 mL (CH_3_)_2_SO. A critical examination of Table 4 designates that the compounds demonstrated improved radical scavenging capabilities. Inhibitory values usually reflect extent of radical scavenging capabilities. The ligands significantly presented percentage inhibitory values lower or comparable to that of the standard indicating their antioxidant possibilities. The antioxidant capabilities of the latter was enhanced substantially upon complexation to divalent metallic spices. Largely, the heteroleptic complexes presented superior DPPH radical scavenging capabilities to the ligands and comparable to that of the standard.

**Table 4:** Antioxidant data of HL, 2,2′-bipy and their heteroleptic complexes.

**Name**	**Concentration**	**% DPPH radical scavenging ability**
Blank	–	–
HL	Ic_50_	57.96
	Ic_100_	80.66
	Ic_200_	83.23
Bipy	Ic_50_	74.76
	Ic_100_	76.23
	Ic_200_	77.40
[Mn(X)(L)(Y)]	Ic_50_	86.96
	Ic_100_	58.53
	Ic_200_	93.23
[Co(X)(L)(Y)]	Ic_50_	82.26
	Ic_100_	87.06
	Ic_200_	93.83
[Ni(X)(L)(Y)]	Ic_50_	58.36
	Ic_100_	73.23
	Ic_200_	75.76
[Cu(X)(L)(Y)].H_2_O	Ic_50_	82.26
	Ic_100_	84.26
	Ic_200_	93.83
Standard ascorbic acid	Ic_50_	86.26
	Ic_100_	87.66
	Ic_200_	88.67

### Molecular Docking Studies

The 3D crystal structures urate oxidase from *Aspergillus flavus* complexed with uracil (PDB ID: 1WS3) and human haematopoietic cell kinase (PDB code: 2HCK) were acquired from the Protein Data Bank (PDB), (http://www.pdb.org) database. They were retrieved together with their co-crystallized ligands, which were used to validate the docking protocols for the binding sites. The proteins were opened in Discovery studio where the water of crystallization and unwanted protein chains were removed. The structures of the ligands were drawn using MarvinSkech 17.2.6. MMFF94 force field was applied in order to minimize energy of both the proteins and ligand molecules. The prepared compounds were then subjected to interact with each of the receptors through molecular docking using PyRx. The protocol facilitates flexible compound docking for various compound conformers within the rigid receptor. Best conformation for each protein-compound complex was chosen and the interaction was visualized in Discovery Studio. The binding energies of these compounds are shown in Table 5.

**Table 5:** Binding free energies ΔG (kcal mol^−1^) of compounds against drug targets.

**S/N**	**Compounds**	**Antioxidant receptor (2HCK)**	**Antifungal receptor (1WS3)**
		**ΔG (kcal/mol)**	**ΔG (kcal/mol)**
1.	HL	−9.64	−9.74
2.	[Mn(X)(L)(Y)]	−9.69	−10.50
3.	[Co(X)(L)(Y)]	−11.43	−9.53
4.	[Ni(X)(L)(Y)]	−12.94	−8.87
5.	[Cu(X)(L)(Y)].H_2_O	−11.04	−9.39
6.	Bipy	−7.30	−7.79
7.	α-Tocopherol	−11.02	ND
8.	Quercetin	−14.08	ND
9.	Fluconazole	ND	−9.56

*ND, Not determined*.

### Authentication of 2HCK and 1WS3

**Figure 2:**
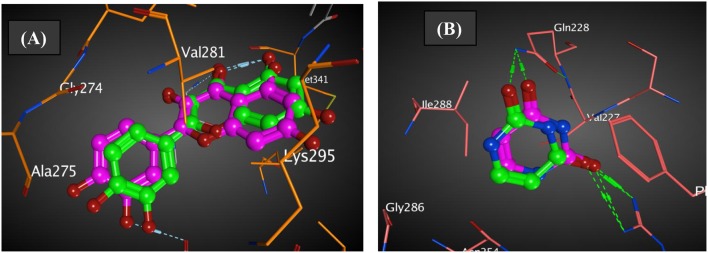
Authentication of docking protocol of **(A)** 2HCK and **(B)** 1WS3 (purple compound, co-crystalized ligand; green compound, docked ligand).

The co-crystallized ligands of 2HCK (quercetin) and 1WS3 (uracil) were retrieved from Pubchem and docked into the binding sites of the drug targets. The results in **Figure 5** presented that the docked ligand (green) superimposed itself on the co-crystallized ligand (purple) in the binding cavity of the receptor. The root mean square deviation (rmsd) amid the docked of quercetin (green) and the co-crystallized quercetin (purple) was 1.51 Å (Figure 2A). Also, the root mean square deviation (rmsd) amid the docked of uracil (green) and the co-crystallized uracil (purple) was 1.49 Å (Figure 2B). The rmsd were <2 Å implying that the ligands were reasonably within the same binding cavity of the receptors as the co-crystallized ligand. Therefore, the docking protocol was capable to yield comparable docking pose of HL with regards to the biological conformation of the same ligand within the crystal assemblage of complex protein, hence our docking protocol was validated.

**Figure 3:**
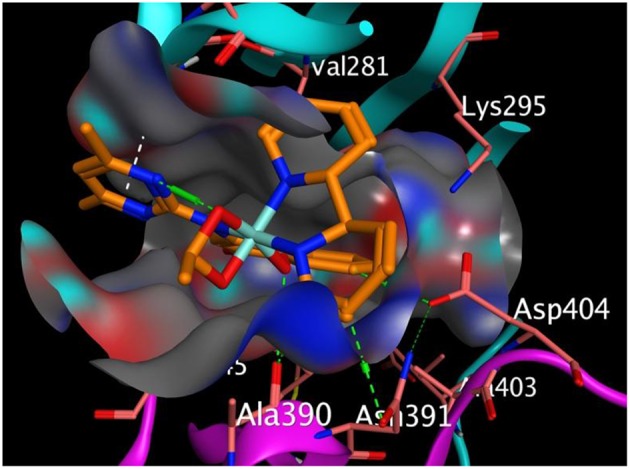
Binding pose of compound 4 in the binding cavity of 2HCK (the green dotted lines signify H-bond; white dotted lines signify pi-H interaction).

**Figure 4:**
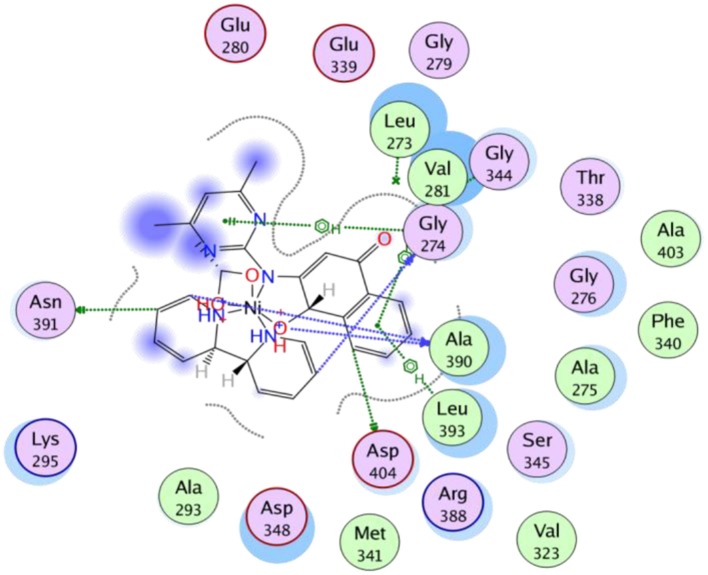
2D representation of the binding interactions of compound 4 with amino acid residues of 2HCK.

Figure 3 represented how compound 4 (with the highest binding affinity to 2HCK among the synthesized compounds) built-in into the binding cavity of the drug target and Figure 4 displayed clearly the 2D chemical interactions of the ligand atoms of 4 with the amino acid residues of the receptor. Hydrogen bonding interactions were noticed between compound 4 and ASP 404, LEU 393, ASN 391, and GLY 274. Other amino acids were involved in other hydrophobic interactions.

**Figure 5:**
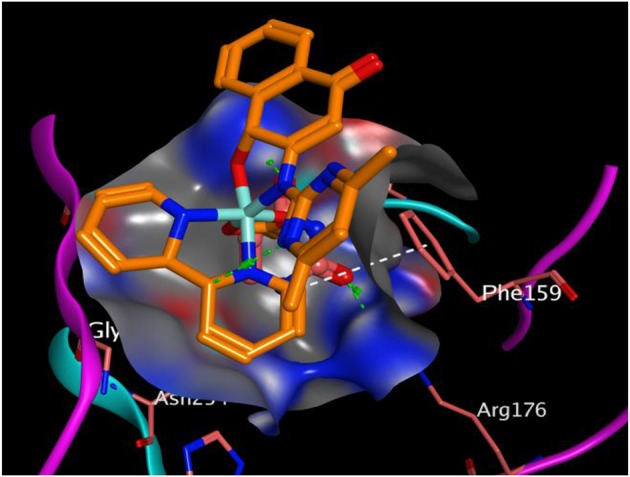
Binding pose of compound 2 in the binding cavity of 1WS3 (the green dotted lines signify H-bond; white dotted lines signify H-pi interaction).

All the synthesized compounds had a comparable binding affinity as the standard drug (fluconazole) against urate oxidase. However, compound 2 showed appreciable binding affinity above the standard drug. The H-pi interaction between the compound 2 and PHE 159 (Figure 5) at a distance of 4.89 Å seem to exhibit substantial role in the binding of the ligands to the receptor. VAL 227, ILE 288, GLN 228, ARG 176, LEU 287, GL Y 286, and ASN 254 interacted hydrophobically with compound 2.

The effective interaction of compounds with the receptor must take place within the receptor's binding sites in order to ellicit the desired pharmacological activity. Hence binding outside the binding sites has no effects. At this binding sites lies different amino acid residues that chemically interact with the atoms of the ligand. We observed that our compounds properly fitted into the binding sites of the receptor when compared with the co-crystallized ligand. The chemical interactions between the π electrons from three different 6-membered aromatic rings of compound 3 with leucine 393 and glycine 274 of 2HCK played greater roles in the observed strong binding affinity (Figure 3).

## Conclusion

On the basis of the analytical and spectral data, the pyrimidinyl ligand, HL acts as bidentate (*N,O*) chelating agent and coordinates to divalent manganese, cobalt, nickel and copper ions through deprotonated secondary amide nitrogen and ketonic oxygen atoms. Experimental (analytical, spectral, and magnetic) data designate the uptake of a six coordinate octahedral stereochemistry for the divalent heteroleptic compounds. The antimicrobial activity of the ligand was enhanced upon complexation with divalent metallic ions, mostly for heteroleptic cobalt complex, with an improved action likened to the ligands and the standard drugs. The divalent heteroleptic complexes exhibited good scavenging activity compared to the standard drug used. *In silico*, the synthesized compounds presented substantial binding attraction with urate oxidase and human haematopoietic cell kinase enzymes.

## Data Availability Statement

The datasets generated for this study can be found in the Protein Data Bank (https://www.rcsb.org) under the accession numbers P08631 and Q00511.

## Author Contributions

CF designed the work. CF, SO, and AE carried out the experiment and wrote up the work.

### Conflict of Interest

The authors declare that the research was conducted in the absence of any commercial or financial relationships that could be construed as a potential conflict of interest.
